# Phosphate and ATP uptake by lake bacteria: does taxonomical identity matter?

**DOI:** 10.1111/1462-2920.13368

**Published:** 2016-06-08

**Authors:** Carina Rofner, Ruben Sommaruga, María Teresa Pérez

**Affiliations:** ^1^ Lake and Glacier Ecology Research Group, Institute of Ecology, University of InnsbruckTechnikerstraße 256020InnsbruckAustria

## Abstract

Phosphorus often limits bacterial production in freshwater ecosystems. However, little is known on whether different bacteria contribute to inorganic and organic phosphorus uptake proportionally to their relative abundance and production. Here, we followed the temporal dynamics of the main heterotrophic bacterial taxa taking up inorganic phosphate (^33^P‐Pi) and organic phosphorus (^33^P‐ATP) in two mountain lakes and compared them to their contribution to bacterial production (^3^H‐leucine uptake). The short turnover times for Pi and ATP suggested that in both lakes, phosphorus was limiting most of the year. The bulk uptake rates and the fractions of cells labelled positive for Pi and ATP uptake followed a seasonal trend with minima in winter and maxima in summer. Generally, the bacterial taxa examined contributed to Pi and ATP uptake proportionally to their relative abundance, but not always to their contribution to bacterial production. For instance, AcI *Actinobacteria* were often underrepresented in phosphorus uptake compared with leucine incorporation suggesting they might have high intracellular C:P ratios. Our results emphasize that ATP utilization is widespread among freshwater bacteria and indicate that members within the dominant bacterial taxa (*Actinobacteria* and *Betaproteobacteria*) have variable phosphorus requirements, probably due to their different growth potential and variable degrees of homeostasis.

## Introduction

Phosphorus is an essential nutrient for life (DeDuvo, 1991; Karl, 2000) that is known to limit both primary and bacterial secondary production in oligotrophic marine and freshwater ecosystems (Carlson, 1977; Vadstein, 2000). Although inorganic phosphate (Pi) is easily taken up by microbes (Björkman and Karl, 1994; Karl, 2000), the uptake of labile dissolved organic phosphorus (DOP) is an alternative strategy to cope with low Pi concentrations.

In aquatic ecosystems, DOP accounts for ca. 50%–80% of total dissolved phosphorus (TDP) (Minear, 1972; Ammerman *et al*., 2003; Lomas *et al*., 2010) and consists mainly of phosphonates and phosphoesters (Kolowith *et al*., 2001). Among DOP compounds, labile phosphoesters such as ATP have been found to significantly decrease the Pi demand of the plankton community (Berman, 1985, 1988; Björkman and Karl, 1994). However, those pioneering studies did not resolve which microorganisms efficiently use this phosphoester or whether there are any differences in Pi and ATP uptake among phyla.

More recent studies using flow cytometry combined with cell‐sorting quantified the uptake rates of sorted communities of heterotrophs and small autotrophs (Zubkov *et al*., 2007; Casey *et al*., 2009; Duhamel *et al*., 2012) and showed, for example, that in the Sargasso Sea, Pi and ATP uptake were dominated by heterotrophic bacteria which comprised the majority of the microbial community (Michelou *et al*., 2011). Other studies combining microautoradiography with catalysed reporter deposition fluorescence *in situ* hybridization (MAR‐CARD‐FISH) found that the contribution of individual marine bacterial taxa to Pi (Longnecker *et al*., 2010) and ATP uptake (Alonso‐Sáez and Gasol, 2007) differed depending on the season and system considered. The latter authors further showed that the proportions of different Alphaproteobacterial phylotypes to ATP uptake varied greatly, suggesting that different members within a phylum or class exhibit different affinities for P resources.

Previous work in a P‐limited alpine lake (Pérez and Sommaruga, 2006) indicates that fast growing bacterial clades might outcompete other phylogenetic taxa in P acquisition. In fact, Pérez and Sommaruga (2006) observed that the addition of a P‐rich algal‐extract translated into high bacterial production rates and the dominance of the bacterial assemblage by the R‐BT cluster of *Betaproteobacteria* (up to 80% of DAPI‐stained cells). Therefore, in this study we tested the hypothesis that individual bacterial taxa take up inorganic and organic P compounds proportionally to their contribution to bacterial production, and determined whether this contribution changes with lake trophic status and season.

In two mountain lakes of contrasting trophic status, we assessed Pi, ATP (as a model compound for DOP) and leucine uptake (as a proxy for bacterial production) by the most abundant bacteria at the lineage/cluster level (AcI lineage of *Actinobacteria* and R‐BT cluster of *Betaproteobacteria*, a lineage of the genus *Limnohabitans*)) and of less abundant bacteria at the phylum/class level (*Bacteroidetes*, *Alphaproteobacteria*) using MAR‐CARD‐FISH. Since temperature and nutrient availability affect bacterial production (Shiah and Ducklow, 1994) and P uptake rates (Stets and Cotner, 2008), we examined the substrate uptake patterns during the lake stratification in summer and after the autumn lake overturn. To obtain background information on the temporal dynamics of P in the lakes, we further estimated the Pi and ATP bioavailable concentrations and measured bulk substrate uptake rates by the entire microbial community.

## Results

### Lake nutrient concentrations and bacterial community composition

**Figure 1:**
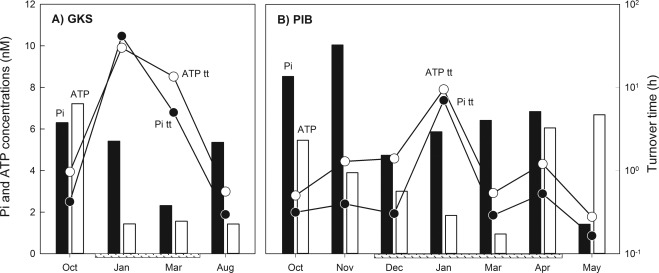
Temporal dynamics of bioavailable Pi and ATP concentrations (black and light bars respectively) and the respective turnover times (tt) of Pi (filled circles) and ATP (open circles). The box underneath the x‐axis represents the ice‐covered period. Note that the *y*‐axis for turnover time is in logarithmic scale.

In GKS, DOC concentrations ranged from 18 to 40 µM, whereas in PIB they were one order magnitude higher (Table 1). TDP concentrations were low in both lakes, but were most often close to the detection limit (< 29 nM) in GKS (Table 1). In both lakes, the estimated bioavailable Pi and ATP concentrations never exceeded 10 nM (Fig. 1). In GKS (Fig. 1A), bioavailable Pi concentrations declined slowly from October till March (from 6.3 to 2.3 nM) and increased again in August (5.4 nM). By contrast, ATP concentrations were high in October (7.3 nM), but remained low during the rest of the year (average 1.5 ± 0.08 nM). In PIB (Fig. 1B), Pi concentration ranged from 5 to 10 nM except for May when it was 1.4 nM. From October till March, ATP concentrations decreased steadily (from 5.5 to 1 nM), but increased again in April and May (Fig. 1B). Bacterial abundance was similar at both depths in GKS (∼6.9*10^5^) and PIB (∼2.4*10^6^) in October, decreased markedly in January when both lakes were ice‐covered (Table 1), but increased again during the summer months.

**Table 1:** Summary of the physico‐chemical and biological conditions found in lakes GKS and PIB.

								Uptake rate (nmol l^−1^ d^−1^)		
Lake	Date	Depth (m)	Temp (°C)	pH	TDP(nM)	DOC(µM)	BA(cells ml^−1^)	Leucine	Pi	ATP
GKS	10.10.2012	1	7.0	7.30	19	18	6.8*10^5^	2.697	0.673	2.438
		8	7.0	7.29	19	23	7.0*10^5^	2.697	0.699	2.070
	22.01.2013	1	1.0	7.33	29	40	1.6*10^5^	0.043	0.014	0.028
	21.03.2013	1	0.9	7.58	19	19			0.220	0.178
	01.08.2013	1	14.3	7.54	26	26	5.8*10^5^	3.556	1.686	3.181
		8	8.0	7.61	42	22	8.3*10^5^	2.131	1.904	3.807
PIB	17.10.2012	1	12.9	7.55	77	207	2.1*10^6^	1.548	0.964	3.193
		15	8.2	7.01	84	184	2.6*10^6^	1.623	0.561	0.780
	06.11.2012	1	8.7	7.33	77	186				
	03.12.2012	1	5.3	6.96	87	188				
	22.01.2013	1	1.8	6.91	94	190	5.6*10^5^	2.182	0.169	0.337
	06.03.2013	1	1.1	7.14	68	152			2.485	2.634
	03.04.2013	1	3.0	7.12	65	146			1.890	2.003
	16.05.2013	1	14.0	7.96	94	182	3.6*10^6^	3.154	1.952	5.359
		15	4.7	7.35	103	156	2.4*10^6^	2.613	1.810	4.509

Temp, Temperature; TDP, Total dissolved phosphorus; DOC: Dissolved organic carbon; BA: Bacterial abundance.

In general, the bacterial community composition in both lakes was dominated (as determined by CARD‐FISH) by the AcI lineage of *Actinobacteria*, followed by *Betaproteobacteria*, *Bacteroidetes*, and *Alphaproteobacteria* (Table 2). In October, when the water column in GKS was mixed, the bacterial community structure was similar at 1 and 8 m depth. Although PIB was still stratified, we did not detect differences in bacterial community composition between the two depths sampled (*t*‐test, *n* = 45, *P* = 0.36). The relative abundance of AcI *Actinobacteria* increased markedly in GKS in August. Similarly, in PIB in May, the relative abundance of AcI *Actinobacteria* increased substantially in the epilimnion, but not at 15 m depth. In GKS, the relative abundance of *Betaproteobacteria* increased from ∼21% in October to ∼32% of DAPI counts in August, whereas in PIB, they always accounted for ∼14% of DAPI counts (Table 2). In GKS, between 43% and 85% of the *Betaproteobacteria* were R‐BT cells, whereas in PIB, this cluster represented ∼38% of *Betaproteobacteria* at 1 m depth, but ∼23% of *Betaproteobacteria* at 15 m depth. *Bacteroidetes* represented ∼15% and 10% of DAPI counts in GKS in October and August, respectively, whereas in PIB their relative abundance was ≤ 5% of DAPI counts. The relative abundance of *Alphaproteobacteria* in both lakes was generally low (≤ 5% of DAPI counts), except for October in GKS where they reached ∼16% of DAPI counts.

**Table 2:** Structure of the bacterial communities in GKS and PIB at dates when MAR‐CARD‐FISH analyses were done.

Lake	Date	Depth (m)	EUBI‐II‐III	ALF968	BET42a	R‐BT065	CF319a	AcI‐852
GKS	10.10.2012	1	75.31 ± 3.32	16.08 ± 1.48	23.27 ± 2.12	10.01 ± 1.85	15.01 ± 3.10	24.57 ± 0.09
		8	77.91 ± 3.18	15.92 ± 2.48	19.74 ± 1.78	11.24 ± 1.66	16.42 ± 1.23	25.34 ± 0.16
	01.08.2013	1	93.92 ± 0.54	3.11 ± 0.34	29.06 ± 0.26	24.59 ± 2.33	8.82 ± 0.36	53.31 ± 2.87
		8	91.03 ± 2.64	2.51 ± 0.35	35.91 ± 2.94	29.10 ± 3.68	10.77 ± 1.74	42.54 ± 0.88
PIB	17.10.2012	1	54.08 ± 1.28	5.16 ± 1.20	12.92 ± 0.74	4.64 ± 0.31	2.99 ± 0.11	21.34 ± 2.32
		15	56.14 ± 4.39	2.85 ± 1.11	12.98 ± 2.26	2.60 ± 0.16	3.56 ± 0.51	24.30 ± 1.79
	16.05.2013	1	67.73 ± 1.27	3.11 ± 0.64	15.72 ± 2.33	6.34 ± 1.35	4.63 ± 1.48	41.36 ± 1.47
		15	68.42 ± 7.61	4.06 ± 0.79	14.19 ± 1.63	3.60 ± 0.23	5.24 ± 0.71	25.79 ± 0.62

EUBI‐II‐III (*Eubacteria*), ALF968 (*Alphaproteobacteria*), BET42a (*Betaproteobacteria*), R‐BT065 (R‐BT cluster of *Betaproteobacteria*), CF319a (*Bacteroidetes*), and AcI‐852 (AcI lineage of *Actinobacteria*). The mean relative abundance of probe‐specific hybridized cells (*n* = 9) is given as % of DAPI counts ± SD.

### Bulk uptake rates and turnover times

Phosphate and ATP uptake rates showed a strong seasonality in GKS (Table 1) with a minimum in January and March and higher values during the rest of the year. This pattern translated into turnover times < 1 h for Pi and ATP in October and August, but as high as 30–40 h during the ice‐covered period (Fig. 1A). Similarly, leucine uptake rates were high in October and August (mean 2.77 ± 0.59 nmol l^−1^ d^−1^), but about two magnitudes lower in January (Table 1). In PIB, leucine uptake rates increased from October onwards during the whole sampling period (Table 1), whereas Pi and ATP uptake rates followed a seasonal trend with a minimum in January and higher rates during the rest of the year (Table 1). Consequently, Pi and ATP turnover times were low throughout the year (Pi 10–32 min; ATP 17–84 min), with the longest turnover times for both substrates found in January (Fig. 1B). In both lakes, water temperature significantly correlated with P uptake rates (Pearson correlation coefficient *r*_Pi_ = 0.75 and *r*_ATP_ = 0.78).

### Proportions of cells taking up Pi, ATP and leucine

**Figure 2:**
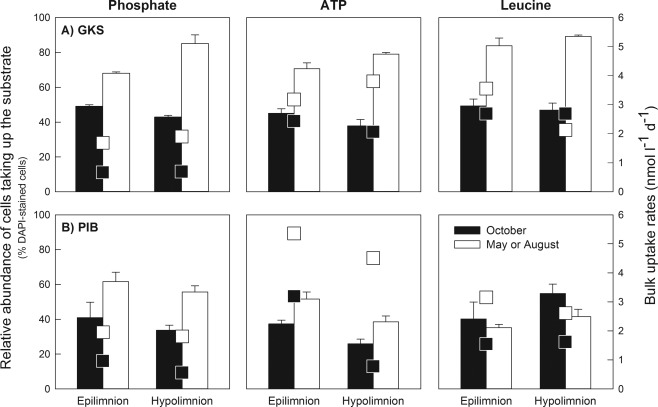
Relative abundance of cells (% DAPI‐stained cells) taking up phosphate, ATP and leucine in the epilimnion and hypolimnion of GKS (A) and PIB (B). Filled bars represent October samples in both lakes. Open bars correspond to May and August samples in PIB and GKS respectively. Squared symbols indicate bulk uptake rates of the substrates in the respective month. Values are means of triplicate samples ± 1SD.

The relative abundance of cells incorporating Pi, ATP and leucine, generally followed the seasonal trend found for the bulk uptake rates in both lakes (Fig. 2; Table 1). Thus, the percentage of cells incorporating Pi and ATP increased significantly from October till May (PIB: *t*‐test, *n* = 10, *P* < 0.001) or August (GKS: *t*‐test, *n* = 10, *P* < 0.001). In GKS, between 38% and 49% of DAPI‐stained cells took up Pi and ATP in October at both depths (Fig. 2A), whereas in August 79%–85% DAPI‐stained cells took up these substrates at 8 m depth and about 68%–71% DAPI‐stained cells at 1 m depth. The percentage of cells incorporating Pi and ATP in PIB ranged from 26% to 41% of DAPI‐stained cells in October and increased by one third in May (Fig. 2B). The proportions of cells positive for leucine uptake increased markedly from October to August in GKS, but remained constant or slightly decreased in PIB (Fig. 2B). In both lakes, the Pi and ATP bulk uptake rates doubled from October to May (PIB) and August (GKS). Similarly, the leucine uptake rates increased by one third in PIB and in the epilimnion of GKS (Fig. 2).

### Dynamics of substrate uptake by individual bacterial taxa

**Figure 3:**
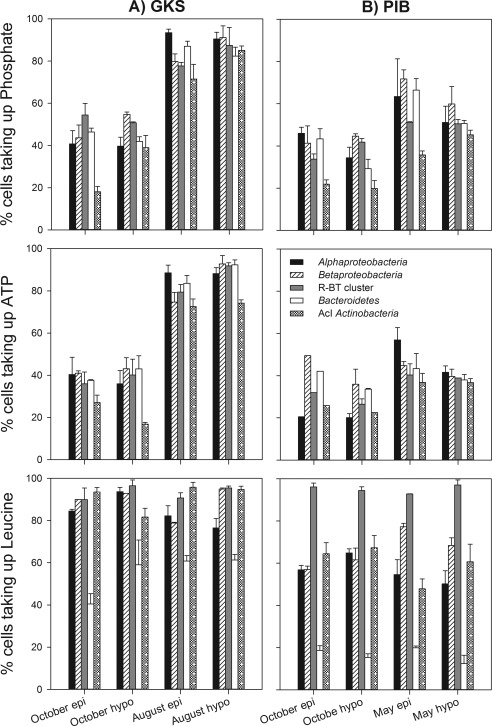
Percentage of *Alphaproteobacteria*, *Betaproteobacteri*a, R‐BT cluster of *Betaproteobacteria*, *Bacteroidetes*, and AcI *Actinobacteria* taking up phosphate, ATP, and leucine in GKS (A) and PIB (B). Values are the mean of triplicate samples ± 1SD collected in the epilimnion (epi) and hypolimnion (hypo) of both lakes in October and in May (PIB) or in August (GKS).

The bacterial taxa examined differed greatly in their contribution to Pi and ATP uptake depending on the month considered (Fig. 3), whereas the proportions of leucine‐positive cells remained fairly constant between October and May (PIB) or October and August (GKS). Nonetheless, some striking patterns in Pi, ATP and leucine uptake were found within certain taxa.

For instance, a lower percentage of cells belonging to the AcI lineage of *Actinobacteria* incorporated either Pi or ATP when compared with other bacterial taxa examined in both lakes (Fig. 3), whereas their contribution to leucine uptake was similar to that of other taxa (∼60% and ∼90% of hybridized cells in PIB and GKS, respectively). By contrast, bacteria of the phylum *Bacteroidetes* were underrepresented in the uptake of leucine in both lakes (< 61% of hybridized cells). Thus, the proportions of *Bacteroidetes* cells positive for Pi and ATP uptake were significantly higher than for leucine uptake (*t*‐test; different means: ATP *P* < 0.02, Pi *P* < 0.03). By contrast, the R‐BT cluster of *Betaproteobacteria* showed on average 95% of hybridized cells positive for leucine uptake in both lakes. This cluster generally reflected the Pi and ATP uptake patterns observed at the class level and accounted for similar or slightly lower proportions positive in the uptake of these compounds than *Betaproteobacteria*. Both, *Alpha*‐ and *Betaproteobacteria* showed a substantial increase in the proportions of cells that were labelled positive for Pi and ATP uptake from October (range: 20%–55% of hybridized cells) to May (range PIB: 40%–72% of hybridized cells) or August (range GKS: 75%–93% hybridized cells), whereas > 77% and > 50% of hybridized cells were labelled positive for leucine uptake in GKS and PIB respectively.

### Contribution of bacterial taxa to Pi and ATP uptake compared with their contribution to bacterial abundance and leucine incorporation

The contribution of a specific bacterial taxon to the uptake of Pi and ATP was generally proportional to its contribution to bacterial abundance (Supporting Information Fig. S1). The only exception was for AcI *Actinobacteria* that tended to be underrepresented for Pi uptake in PIB (Supporting Information Fig. S1B). AcI *Actinobacteria*, *Betaproteobacteria* and its R‐BT cluster were overrepresented in the uptake of leucine in GKS in October, and in May in PIB.

**Figure 4:**
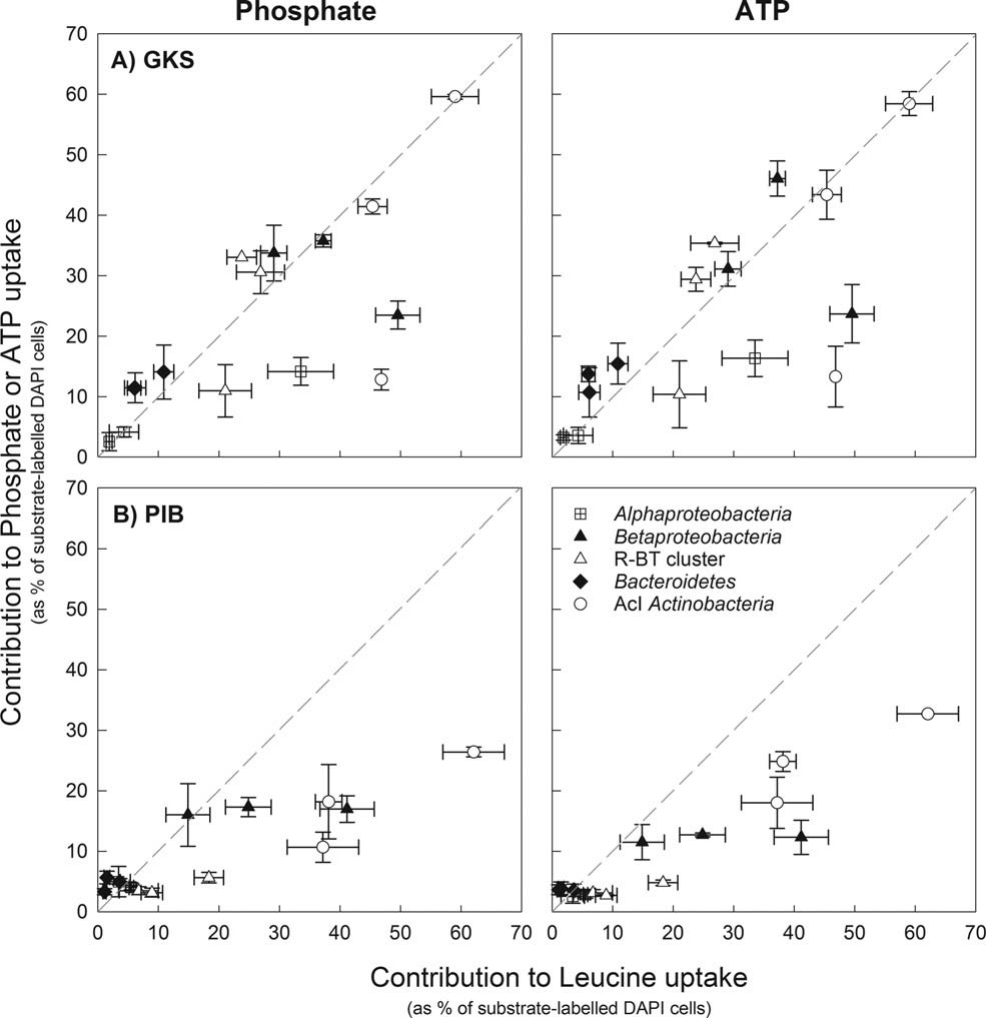
Relative contribution of *Alphaproteobacteria*, *Betaproteobacteria*, R‐BT cluster of *Betaproteobacteria*, *Bacteroidetes* and AcI *Actinobacteria* to phosphate or ATP uptake plotted against their contribution to leucine uptake in GKS (A) and PIB (B). Mean values are given as % of substrate‐labelled DAPI cells ± 1SD. The dashed line indicates a 1:1 relationship. Samples were taken in October 2012 and in May (PIB) or in August 2013 (GKS). For October, epilimnion and hypolimnion values are pooled together (*n* = 5), whereas for May or August, values for both depths are plotted (*n* = 10).

In GKS in August, the contribution of the bacterial taxa examined to Pi or ATP uptake was equal to its contribution to leucine incorporation (Fig. 4A). Whereas in October, they were underrepresented in P uptake when compared with leucine incorporation, except for *Bacteroidetes* (Fig. 4A). In PIB (Fig. 4B), AcI *Actinobacteria* were always underrepresented in Pi and ATP uptake when based on leucine bacterial production, whereas *Alphaproteobacteria* and *Bacteroidetes* contributed to P uptake as expected from their contribution to leucine incorporation. *Betaproteobacteria* and its R‐BT cluster either contributed equal to Pi and ATP uptake when based on leucine incorporation or they were underrepresented.

## Discussion

In aquatic ecosystems, utilization of DOP compounds by bacteria might be an important strategy to cope with scarcity in inorganic P (Berman, 1988; Björkman and Karl, 1994; Sebastián *et al*., 2012). Indeed, our results show for the first time that in P‐limited lakes different bacterial taxa take up both inorganic and organic P (Fig. 3), and contribute to the uptake of these compounds proportionally to their *in situ* abundance (Supporting Information Fig. S1), but not always in relation to their contribution to bacterial production (assessed as cells positive for leucine uptake). Thus, we could show that members within the dominant bacterial taxa in these lakes (AcI lineage of *Actinobacteria* and R‐BT cluster of *Betaproteobacteria*) might have different P requirements probably due to their different growth potential and life strategies. However, we have to note that the findings presented here may reflect the characteristics of the dominant bacterial populations within the targeted lineage and cluster.

### Pi and ATP in situ dynamics and bulk uptake rates

The estimated bioavailable Pi and ATP concentrations, as well as their turnover times, were generally in the range of hours to minutes, and according to Prepas (1983) hint to the bacterial community of both lakes being P‐limited during the study period. The Pi and ATP turnover times in both lakes were similar or even lower than estimates done for other marine and freshwater ecosystems (Supporting Information Table S1). Furthermore, we found that Pi and ATP turnover times, as well as bulk substrate uptake rates followed a marked seasonality certainly due to temporal fluctuations in nutrient concentrations and temperature as found by others (Prepas, 1983; Tanaka *et al*., 2004; Zubkov *et al*., 2007).

When we compared the bulk uptake rates with previous studies, we found that Pi uptake rates were often lower than those measured in the oligotrophic North Atlantic gyre (Zubkov *et al*., 2007; Casey *et al*., 2009; Michelou *et al*., 2011). On the contrary, the ATP uptake rates were higher than reported for the Mediterranean Sea (Alonso‐Sáez and Gasol, 2007) or the North Atlantic gyre (Zubkov *et al*., 2007; Michelou *et al*., 2011), but similar to rates found in the North Pacific subtropical gyre (Björkman and Karl, 2005). Those and our results indicate that DOP utilization by bacteria in oligotrophic systems is widespread (Alonso‐Sáez and Gasol, 2007; Pérez *et al*., 2015) and could be advantageous because DOP compounds such as ATP simultaneously serve as a P‐, C‐ and N‐source. However, as P uptake is a concentration‐dependent process (Cotner and Wetzel, 1992), discrepancies in the uptake rates are certainly not only due to differences among study systems, but are also due to the substrate concentrations used. For instance, here we used four times more ATP than Pi to mimic the fact that in lakes DOP often represents a greater fraction of the P pool compared with inorganic P [reviewed in Chróst (1994)]. A direct comparison of Pi and ATP uptake rates done at the same radiotracer concentrations during the bioassay experiments showed that Pi uptake rates were slightly higher than ATP ones in GKS and up to four times higher in PIB, which can be also deduced from the Pi and ATP turnover times (Fig. 1). This is not surprising because Pi is directly taken up by specific and unspecific transport systems (van Veen, 1997), whereas phosphoesters such as ATP have to be cleaved prior to cell uptake (Cotner and Wetzel, 1991).

### Substrate uptake by individual bacterial taxa

Independently of the taxonomic resolution level used (Figs. 2 and 3), the proportions of cells positive for Pi and ATP uptake were low in both lakes in October, whereas significantly higher proportions of cells took up these substrates in PIB in May and in GKS in August. Year‐round variability in the proportions of cells taking up either Pi or ATP has been observed in marine systems previously (Alonso‐Sáez and Gasol, 2007; Longnecker *et al*., 2010). However, unlike these studies, we found that the contribution of specific freshwater bacterial taxa to Pi or ATP uptake was proportional to their relative abundance (Supporting Information Fig. S1). Pérez *et al*. (2015) observed a similar pattern for ATP uptake in two alpine lakes and thus, this seems to be a recurrent feature in P‐limited mountain lakes. However, the correlation between the relative cell abundance and contribution to P uptake was weaker in the mesotrophic subalpine lake (Supporting Information Fig. S1B), suggesting that this pattern weakens with increasing lake productivity.

In both lakes, the microbial assemblage was dominated by *Actinobacteria* and *Betaproteobacteria* as previously reported for mountain lakes in the central Alps (Warnecke *et al*., 2005; Pérez and Sommaruga, 2006; Salcher *et al*., 2008). Members of these two bacterial taxa are known to differ in several physiological characteristics as for example, in their growth potential. For instance, Šimek *et al*. (2006) found that *Betaproteobacteria*, and particularly their R‐BT cluster (a lineage of the genus *Limnohabitans*), exhibit the highest maximum growth rates among freshwater bacteria, whereas *Actinobacteria* have moderate maximum growth rates. According to the growth rate hypothesis (Elser *et al*., 2000), faster growing organisms might have high P requirements due to the increase of P‐rich cell structures needed for growth [e.g., ribosomes (Franklin *et al*., 2011)] and betaproteobacterial phylotypes often overgrow other bacteria when the concentration of inorganic or organic P increases (Burkert *et al*., 2003; Šimek *et al*., 2005; Hornák *et al*., 2006; Posch *et al*., 2007). Thus, here we expected fast growing bacteria such as the R‐BT cluster of *Betaproteobacteria* to be preponderant in P acquisition. However, when we compared the contribution of the different bacterial taxa to Pi and ATP uptake versus their contribution to leucine incorporation (as a proxy for bacterial production), we observed different patterns depending, not only, on the bacterial taxa, but also on the lake and season considered.

For instance, in the oligotrophic alpine lake GKS in August, all bacterial taxa examined contributed to Pi and ATP uptake as expected based on leucine incorporation. However, in October, *Betaproteobacteria*, its R‐BT cluster, *Alphaprotebacteria*, and AcI *Actinobacteria* were underrepresented in P uptake when compared with their contribution to bacterial production (Fig. 4A). During the autumn lake overturn, nutrient‐rich deep waters mix with surface ones and bacteria may have access to a larger pool of bioavailable P as indicated by the higher bioassayed Pi and ATP concentrations in October (Fig. 1). Additionally, bacteria in October might have accumulated polyphosphates, and so reducing their demand for Pi and ATP. This was shown in the study of Sebastián *et al*. (2012) where P‐limited heterotrophic bacteria accumulated polyphosphates after a Pi amendment in oligotrophic marine waters. An alternative explanation is that the bacterial assemblage in October was dividing at a slower rate than in August, and thus their P demand for DNA replication was reduced (Neidhardt and Magasanik, 1960). However, the pronounced differences obtained in Pi and ATP uptake versus leucine bacterial production between lakes and seasons may be also attributed to changes in the composition of dominant bacteria within the targeted taxa. For instance, the R‐BT cluster in GKS was clearly responsible for the substrate uptake patterns found at the class level which is reasonable because they represented a substantial fraction of *Betaproteobacteria*. However, in PIB, the R‐BT cells were only a minor fraction of *Betaproteobacteria* and thus, other Betaproteobacterial phylotypes (Salcher *et al*., 2008) contributed as well to the dynamics observed. This is particularly true for leucine uptake where the patterns observed at the class and at the cluster level clearly differed (Fig. 3). Similarly, it is likely that different clades within AcI *Actinobacteria* were responsible for the seasonal and the observed lake‐specific Pi and ATP uptake patterns. For instance, Warnecke *et al*. (2005) found that different AcI clades prevail in GKS and PIB. Different temperature and pH optima, but also biogeographic patterns seem to explain Actinobacterial clade distribution (Hahn and Pöckl, 2005; Newton *et al*., 2007).

Unlike *Betaproteobacteria*, the dominant lineage in both lakes AcI *Actinobacteria*, was often underrepresented in the uptake of Pi and ATP when compared with other bacterial taxa examined. Given the numerical dominance of AcI *Actinobacteria* in freshwater habitats (Pernthaler *et al*., 2001; Warnecke *et al*., 2005) and their high contribution to bacterial production (Warnecke *et al*., 2005; Pérez *et al*., 2010; Salcher *et al*., 2013), one could expect also a substantial contribution of bacteria from this lineage to P uptake. However, they were always underrepresented in Pi and ATP uptake when compared with their contribution to leucine incorporation in PIB and in autumn in GKS. This might indicate that members of the AcI lineage have low P requirements as it has been shown in the oligotrophic Mediterranean Sea for the most abundant taxa, the alphaproteobacterial clade SAR11 (Sebastián *et al*., 2012). In this study, the proportions of SAR11 cells taking up Pi and ATP decreased immediately upon Pi amendment, whereas the proportions of other bacterial taxa positive for P uptake kept constant. The authors suggested that cells from this clade did not follow the strategy of ‘luxury uptake’ to store P in polyphosphates, but rather exhibited reduced P requirements. The high abundance of AcI *Actinobacteria* in freshwaters might be due to a similar strategy to that of SAR11 in marine waters, finding the right balance between defensive and competitive abilities by reducing their requirements for the limiting nutrient (Thingstad *et al*., 2014).

Rapid growing cells have a higher P content than slow growing ones (Sterner, 1995), and *Actinobacteria* have lower maximum growth rates compared with other bacterial taxa (Šimek *et al*., 2006). This might explain their apparent lower P requirements. The study of Scott *et al*. (2012) showed that the Actinobacterial *Arthrobacter* sp. exhibits weak homeostasis and keeps high cellular C:P ratios during P‐deficient and –sufficient conditions. A high C:P ratio could explain the proportionally high contribution of AcI *Actinobacteria* to leucine incorporation as compared with P uptake (Fig. 4). Furthermore, it could explain their prevalence during periods of P‐depletion, and their disadvantage during nutrient pulses, when they are outcompeted by faster‐growing cells such as the R‐BT cluster (Pérez and Sommaruga, 2006; Šimek *et al*., 2006).

In contrast to all other bacterial taxa examined, *Bacteroidetes* contributed slightly more to Pi and ATP uptake than expected based on their contribution to bacterial production. This result is only partly surprising because *Bacteroidetes* have been found to be underrepresented in leucine uptake (Hornák *et al*., 2006; Longnecker *et al*., 2010; Pérez *et al*., 2010; Sebastián *et al*., 2012), but to contribute substantially to Pi or ATP uptake (Alonso‐Sáez and Gasol, 2007; Sebastián *et al*., 2012; Pérez *et al*., 2015). Longnecker *et al*. (2010) suggested that Pi might be a more appropriate bacterial activity marker than leucine. However, our results support this statement only partly, considering for example, that in our study AcI *Actinobacteria* were often underrepresented in Pi uptake when compared with leucine incorporation (> 50% of hybridized cells). Thus, we argue that the use of one single marker, either ^3^H‐leucine or ^33^P‐Pi, is insufficient to correctly assess the metabolic activity of all bacterial taxa simultaneously. In any case, our findings indicate that members of freshwater *Bacteroidetes* play a non‐negligible role in the cycling of low‐molecular‐weight inorganic and organic P compounds.

## Conclusions

Although substrate partitioning between bacterial taxa has been reported previously in marine (Alonso‐Sáez and Gasol, 2007; Longnecker *et al*., 2010; Sebastián *et al*., 2012) and freshwaters (Buck *et al*., 2009; Salcher *et al*., 2013; Pérez *et al*., 2015), our results indicate that under P‐limiting conditions, many bacterial taxa contribute to the uptake of inorganic and organic P proportionally to their *in situ* abundance. However, when based on leucine incorporation, our findings showed that certain bacteria within dominant taxa follow different strategies to cope with low P concentrations. The proportionally lower contribution of AcI *Actinobacteria* to P uptake as compared with bacterial production suggests that this lineage might have high cellular C:P ratios and thus, lower P requirements than other freshwater bacteria helping to explain their prevalence in P‐limited systems. In contrast, the R‐BT cluster of *Betaproteobacteria* seems to favour a strong homeostasis during P‐deficiency and is able of both, to dominate at nutrient pulses overgrowing other bacteria and to outlast P‐depleted conditions probably through cytoplasmic P storage.

## Experimental procedures

### Study sites description and sample collection

Samples were collected from two mountain lakes in the Austrian Alps, the oligotrophic alpine lake Gossenköllesee (Kühtai, 47° 13′ N 11° 01′ E) and the mesotrophic subalpine lake Piburgersee (Ötztal, 47° 11′ N 10° 53′ E). Gossenköllesee (GKS; 2417 m a.s.l.) is a holomictic dimictic lake (1.7 ha) with a maximum depth of 9.9 m in its central area, where samples were collected. Piburgersee (PIB; 913 m a.s.l.) is a meromictic dimictic lake (13.4 ha) with a maximum depth of 24.6 m and was subject to anthropogenic eutrophication till the 70's when restoration took place (Pechlaner, 1968).

PIB was sampled seven times between October 2012 and May 2013 at approximately monthly intervals, whereas GKS was sampled in four occasions (once per season) from October 2012 till August 2013. In October, GKS was already mixed (7.0°C), whereas PIB was still slightly stratified (Table 1). Water samples were also taken when both lakes were ice‐covered. This period lasted from November until the beginning of July in GKS and from December until April in PIB. At every sampling date, water samples were collected at 1 m depth with a 5 l Schindler‐Patalas sampler with an integrated thermometer. The water collected (2 l) was transferred to pre‐combusted (450°C, 4 h) borosilicate glass flasks and used to bioassay Pi and ATP concentrations, to assess dissolved organic carbon (DOC) and to estimate bulk uptake rates by the entire bacterial community. An additional 1 l sample was transferred into a clean polyethylene bottle and used for pH and TDP analysis. TDP concentrations were estimated by the molybdenum blue method (Vogler, 1966) and DOC concentrations were analysed as described in Hörtnagl *et al*. (2010). Briefly, a 50 ml subsample was filtered through two pre‐combusted (450°C, 4 h) GF/F filters (Whatman) placed on a stainless steel syringe holder. The filtrate was acidified with hydrochloric acid to pH 2 and DOC concentrations were determined on a Shimadzu TOC‐V_CPH_ series. In each lake, additional water samples for MAR‐CARD‐FISH analyses were collected in two occasions. In GKS, samples from 1 m to 8 m depth were collected in October 2012 after the autumn overturn, and in August 2013 during the summer stratification. In PIB, the epiliminion (1 m) and the hypolimnion (15 m) were sampled in October 2012 and in May 2013 when the lake was still thermally stratified.

### Incubations for microautoradiography (MAR)

For microautoradiography, triplicate samples (20 ml for GKS, 10 ml for PIB) and one formaldehyde‐killed blank (2% final concentration) were incubated in the dark at *in situ* temperature with one of the following three substrates purchased from Perkin Elmer: ^33^P‐Pi (specific activity 155.8 Ci mg^−1^; final concentration 50 pM), ^33^P‐ATP (specific activity 3000 Ci mmol^−1^; final concentration 200 pM) and ^3^H‐leucine (specific activity 56 Ci mmol^−1^; final concentration 20 nM). Incubations lasted 2–3 h in the case of ^33^P‐Pi and ^33^P‐ATP, and 1–2 h for ^3^H‐leucine and were stopped by adding formaldehyde (2% final concentration). Samples were kept overnight at 4°C and filtered on the next day onto 0.22 µm polycarbonate white filters (Millipore GTTP). Filters were rinsed with 5–10 ml of 0.22 µm filtered MQ‐water and stored frozen until further processing.

### Bulk uptake rates by the entire bacterial assemblage

Bulk uptake rates of ^33^P‐Pi, ^33^P‐ATP and ^3^H‐leucine (final concentrations 50 pM, 200 pM, 20 nM, respectively) were determined by measuring the radioactivity incorporated into microbial cells. Duplicate samples (10 ml for GKS, 5 ml for PIB) plus a formaldehyde‐killed blank were inoculated with the radiotracers and incubated as previously described. Incubations lasted for about 30–60 min for ^33^P‐Pi, 45–60 min for ^33^P‐ATP and 1 h for ^3^H‐leucine, and were terminated by adding formaldehyde at 2% final concentration. To minimize isotope leakage, ^33^P‐samples were filtered within 20 min onto 0.22 µm polycarbonate white filters (Poretics), using a Whatman GF/C prefilter soaked in a solution of 100 mM KH_2_PO_4_. Samples incubated with ^3^H‐leucine were filtered through 0.22 µm polycarbonate white filters (Poretics) and extracted with trichloroacetic acid (5%) for 5 min. The radioactivity on the filters was assessed on the next day on a Beckman LS 6000IC scintillation counter.

### MAR‐CARD‐FISH procedure

Samples for CARD‐FISH were processed using the method of Pernthaler *et al*. (2002) as modified by Sekar *et al*. (2003). Briefly, filters sections were hybridized (2 h, 35°C) with the following horseradish peroxidase‐labelled (HRP) oligonucleotide probes (ThermoHybaid) targeting *Bacteria* [EUB I‐II‐III; Daims *et al*. (1999)], *Alphaproteobacteria* [ALF968; Neef (1997)], *Betaproteobacteria* [BET42a; Manz *et al*. (1992)] and its R‐BT cluster [R‐BT065; Šimek *et al*. (2001)], *Bacteroidetes* [CF319a; Manz *et al*. (1996)], and *Actinobacteria* [AcI‐852; Warnecke *et al*. (2005)]. Information on probe‐coverage and outgroup‐hits is found in Amann and Fuchs (2008). Actinobacterial probe AcI‐852 was used instead of HGC69a because better coverage and signal amplification was achieved with this probe (data not shown). After signal amplification with Tyramide Alexa488, filters were cut into two halves. One half was counterstained with 4′,6‐diamidino‐2‐phenylindole (DAPI) and mounted for epifluorescence microscopy and the other half was processed for MAR. In total, 432 individual filter sections were prepared for CARD‐FISH and 360 for MAR‐FISH.

Image acquisition for CARD‐FISH+ cells and DAPI+ cells was done on a semi‐automatic epifluorescence microscope (Zeiss Imager.Z1) equipped with a scanning stage holding eight microscopic slides. Thirty‐six paired images per filter were taken using a 63x magnification with a HCR camera at two excitation wavelengths (DAPI 880nm, Alexa488 488nm). Image processing (image conversion, image quality control, metafile creation) and cell enumeration was done with the freeware ACMEtool2 (Version 2012‐07‐15) of M. Zeder, technology Gmbh. At least 10 well‐shot paired images were used for cell quantification using in most cases the set definitions of the software (DAPI/FISH area < 22 and > 6).

CARD‐FISH filters stained with DAPI were also used to assess bacterial abundance. About 400–1300 homogenously distributed cells per filter were counted manually with a Zeiss Axiophot 2 epifluorescence microscope.

Microautoradiogaphy was done according to Tabor and Neihof (1982) after transferring cells onto cover slips (Cottrell and Kirchman, 2000, 2003). Samples incubated with the ^33^P‐substrates were exposed for 2.5–6 h, whereas samples incubated with ^3^H‐leucine needed 20–48 h exposure. Further details on slide preparation and microscopic examination can be found in Pérez *et al*. (2015).

### Bioassay

Bioavailable Pi and ATP concentrations were measured at 1 m depth in both lakes following the protocol described by Zubkov *et al*. (2007) with some modifications. Briefly, triplicate 1 ml samples and a formaldehyde‐killed blank were incubated with ^33^P‐Pi or ^33^P‐ATP at a standard concentration of 0.2 nM and diluted with non‐labelled KH_2_PO_4_ or ATP to give the following final concentrations 0.2, 0.4, 0.8, 1.6, and 3.2 nM. Every 10 min (for Pi) or every 15 min (for ATP), a subsample was fixed. The samples were filtered onto 0.22 µm polycarbonate filters (Poretics) within 20 min and the filters were covered with scintillation cocktail to assess the radioactivity on a scintillation counter (LS 6000IC). Turnover times were calculated as follows:

Turnover time [h] =−Time [h]/ln(((1−(Rf−Rb))/Rt)

where Rf and Rb are the radioactivity (in DPM) of the filter and the blank respectively, and Rt the total tracer added (Moutin and Thingstad, 2002). Linear regression of the turnover times versus the added substrate concentrations allows to estimate the terms Ks + [*S*] and *v*_max_, where Ks is the half saturation constant, [*S*] is the ambient substrate concentration (either Pi or ATP) and *v*_max_ is the maximum uptake rate. The *y*‐intercept with the regression line gives the turnover time at ambient concentration (zero tracer added), and the *x*‐intercept gives an estimate of the maximum ambient substrate concentration by assuming that at low substrate concentrations Ks is negligible small compared with the *in situ* concentration.

### Statistical analyses

All statistical analyses were run on PAST.exe (Vers.2.17c). A one‐way analysis of variance (ANOVA) or a *t‐*test (two sample test) was used to check for significant differences among sample means of bacterial taxa taking up the substrates. Sample means were compared between seasons, depths, substrates and bacterial taxa. Pearson correlation coefficients were calculated to relate environmental variables to substrate uptake rates. Ordinary Least Squares regression was used to determine the relationship of the bacterial taxa in Pi, ATP and leucine uptake versus their abundance.

## Supporting information

Additional Supporting Information may be found in the online version of this article at the publisher's web‐site:

**Fig. S1**. Relative contribution of *Alphaproteobacteria, Betaproteobacteria*, R‐BT cluster of *Betaproteobacteria* (R‐BT cluster), *Bacteroidetes*, and AcI *Actinobacteria* to phosphate, ATP and leucine uptake plotted against their contribution to bacterial abundance in GKS (A) and PIB (B). Mean values are given as % DAPI‐stained cells +/− 1 SD. The dashed line indicates a 1:1 relationship. Samples were taken in October 2012 and May (PIB) OR August 2013 (GKS). In October, epilimnion and hypolimnion values are pooled together (n = 5), whereas in May or August, values for both depths are plotted (n = 10).

**Table S1.** Summary of phosphate (Pi) and ATP turnover times (tt) in marine and freshwater ecosystems, as well as in mesocosms experiments and in the present study.
