# Supplementary material for: Phosphate and ATP uptake by lake bacteria: does taxonomical identity matter?
Source: Environ Microbiol. 2016 Jun 8;18(12):4782–93. doi: 10.1111/1462-2920.13368 (PMC5213779; doi:10.1111/1462-2920.13368)

Supplementary Figure 1. Relative contribution of *Alphaproteobacteria*, *Betaproteobacteria*, R-BT cluster of *Betaproteobacteria* (R-BT cluster), *Bacteroidetes*, and AcI *Actinobacteria* to phosphate, ATP and leucine uptake plotted against their contribution to bacterial abundance in GKS (A) and PIB (B).

Mean values are given as % DAPI-stained cells +/- 1 SD. The dashed line indicates a 1:1 relationship. Samples were taken in October 2012 and in May (PIB) or August 2013 (GKS). In October, epilimnion and hypolimnion values are pooled together (n=5), whereas in May or August, values for both depths are plotted (n=10).

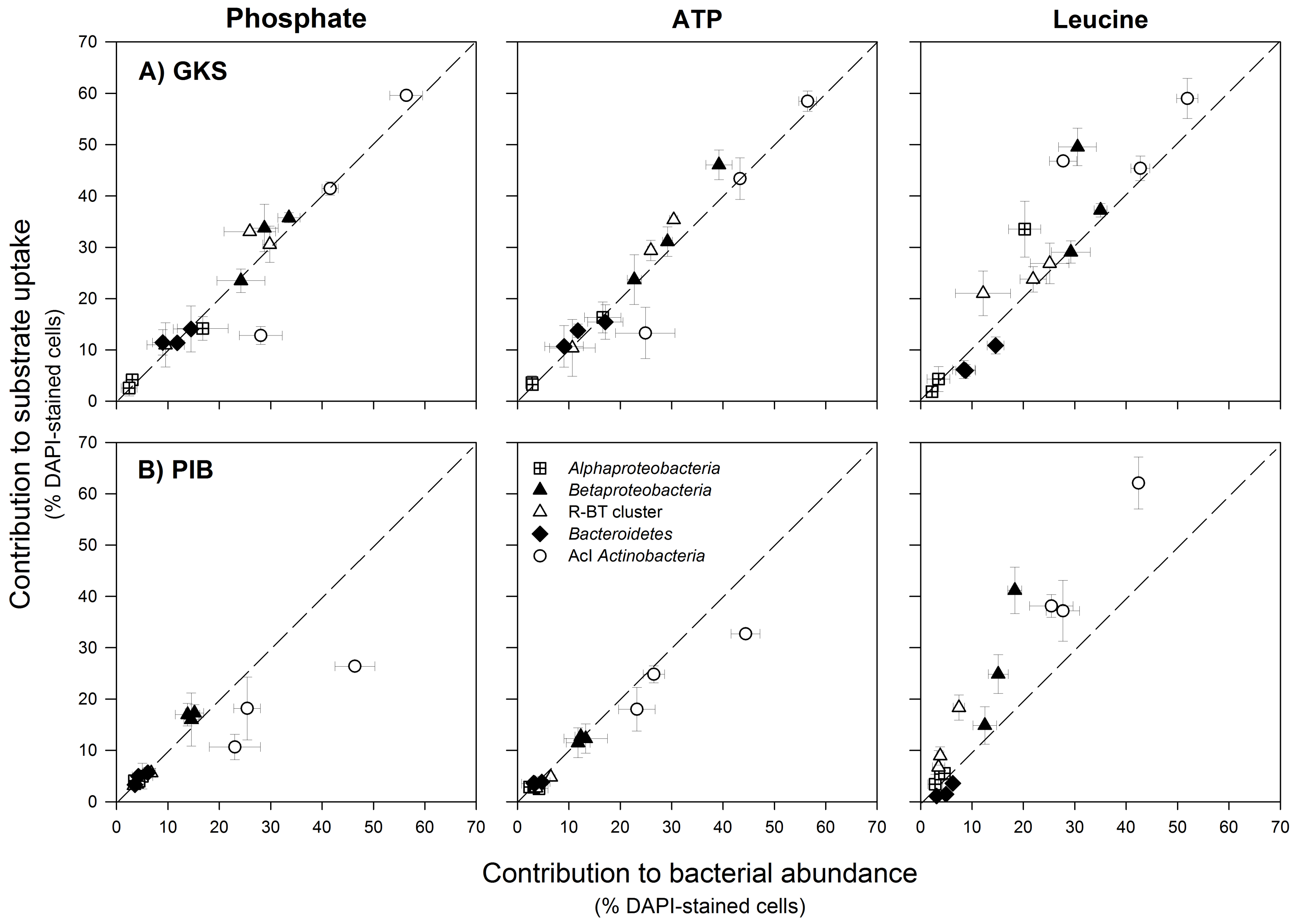

Supplement: Supplementary file 1 — Fig. S1. Relative contribution of Alphaproteobacteria, Betaproteobacteria, R‐BT cluster of Betaproteobacteria (R‐BT cluster), Bacteroidetes, and AcI Actinobacteria to phosphate, ATP and leucine uptake plotted against their contribution to bacterial abundance in GKS (A) and PIB (B). Mean values are given as % DAPI‐stained cells +/− 1 SD. The dashed line indicates a 1:1 relationship. Samples were taken in October 2012 and May (PIB) OR August 2013 (GKS). In October, epilimnion and hypolimnion values are pooled together (n = 5), whereas in May or August, values for both depths are plotted (n = 10). [file EMI-18-4782-s001.PDF]
