# Supplementary material for: Phosphate and ATP uptake by lake bacteria: does taxonomical identity matter?
Source: Environ Microbiol. 2016 Jun 8;18(12):4782–93. doi: 10.1111/1462-2920.13368 (PMC5213779; doi:10.1111/1462-2920.13368)
Supplement: Supplementary file 2 — Table S1. Summary of phosphate (Pi) and ATP turnover times (tt) in marine and freshwater ecosystems, as well as in mesocosms experiments and in the present study. [file EMI-18-4782-s002.pdf]

Supplementary Table 1. Summary of phosphate (Pi) and ATP turnover times (tt) in marine and freshwater ecosystems, as well as in mesocosms experiments and in this study.

Location	Study system	Depth, m	Water column	Pi tt	ATP tt	Reference
Alberta, Canada	8 Lakes	6.2-19.4	Stratified	0.05-0.7 h		Prepas 1983
	10 Lakes	2.9-6.4	Stratified, mixed	0.03-36 h		
Canada	2 Lakes	0-9.5			0.2-1 h	Bentzen *et al*. 1992
Hawaii, USA	Coastline	0.5-1		20-61 h		Björkman & Karl 1994
Mediterranean Sea	9 Stations	~50	mixed	2.9-3711 h		Zohary & Robarts 1998
Mediterranean Sea	Bay	0-30	Stratified-mixed	< 2 h		Tanaka *et al*. 2004
	Bay	30-75	stratified	> 30 h		
Baltic Sea	3 Stations	1-2			5-276 h	Nausch & Nausch 2006
Baltic Sea	Mesocosm			0.02-80 h	0.04-8 h	Løvdal *et al*. 2007
Mediterranean Sea	Mesocosm			1 h	3-6 h	Sebastian *et al*. 2012
Austria	Lake PIB	1	Stratified, mixed	0.2-7 h	0.3-9.5 h	This study
Austria	Lake GKS	1	Stratified, mixed	0.3-42 h	0.6-30 h	This study
